# Funding Programs Relevant to Spinal Cord Injury Research and Their Approaches to Research Partnerships: An Environmental Scan

**DOI:** 10.34172/ijhpm.8813

**Published:** 2026-04-11

**Authors:** Zhiyang Shi, Alanna Shwed, Ian D. Graham, Gayle Scarrow, Peter Athanasopoulos, Vanessa K. Noonan, John Chernesky, Kathryn M. Sibley, SCI IKT Guiding Principles Partnership Panel, Heather L. Gainforth

**Affiliations:** ^1^Department of Kinesiology and Physical Education, McGill University, Montreal, QC, Canada.; ^2^School of Health and Exercise Sciences, University of British Columbia Okanagan, Kelowna, BC, Canada.; ^3^School of Epidemiology and Public Health, University of Ottawa, Ottawa, ON, Canada.; ^4^Michael Smith Health Research BC, Vancouver, BC, Canada.; ^5^Spinal Cord Injury Ontario, Toronto, ON, Canada.; ^6^Praxis Spinal Cord Institute, Vancouver, BC, Canada.; ^7^Department of Community Health Sciences, University of Manitoba, Winnipeg, MB, Canada.

**Keywords:** Integrated Knowledge Translation, Partnered Research, Research Funding, Meaningful Engagement, Canada, United States

## Abstract

**Background:**

Establishing research partnerships can help close the research-practice gap. The Integrated Knowledge Translation Guiding Principles were developed in a spinal cord injury research context as a resource to facilitate research partnerships. Research funders play a significant role in the spinal cord injury research system. However, how funders define, require, evaluate, support research partnerships is rarely reported. This study identified spinal cord injury research funders in Canada and the United States, identified their approaches to supporting research partnerships, and explored organizational perspectives of principles of partnership.

**Methods:**

An environmental scan was conducted through five steps: (1) identifying spinal cord injury research organizations that funded the greatest number of spinal cord injury research publications in Canada and the United States between 2017 and 2022; (2) identifying one funding program related to research partnerships of each funder; (3) extracting online information of the programs; (4) interviewing funder informants; and (5) descriptive and deductive content analysis. The five steps were completed between April 2022 and September 2024. An additional data collection was conducted in July 2025 on a relevant National Institutes of Health funding program.

**Results:**

Sixteen organizations and seventeen partnership-supportive programs were identified. Six programs defined partnerships as researchers and research users engaging throughout the research process. Eleven programs required applicants to describe the partnership in applications and explicitly stated their peer review evaluation criteria. The programs supported research partnerships through remuneration for partners’ engagement (n=6), facilitating connections between researchers and potential partners (n=3), and helping applicants prepare applications (n=4). The programs had few strategies to evaluate awarded partnerships post-grant. Three descriptive categories emerged from the interviews: (1) Varied support for research partnerships; (2) Minimal capacity for partnership evaluation post-grant; and (3) Need for tools and resources to further support research partnerships.

**Conclusion:**

Differences existed in how spinal cord injury research funders in Canada and the United States defined, required, evaluated, and supported research partnerships. The results provided an initial landscape of funders’ role in the spinal cord injury research system and may inform strategic efforts to optimizing meaningful engagement in a broader health research context.

## Background

**Key Messages**

**Implications for policy makers**

- Clarify research partnership definitions and review criteria: Funding organizations should consider developing more explicit definitions and peer review criteria for research partnerships.
- Expand support beyond funding for research partnerships: Funding organizations should consider offering various types of support in addition to research funds, such as assistance with applications, training resources, and remuneration for partners’ engagement.
- Strengthen partnership evaluation post-grant: Funding organizations should consider building greater capacity for ensuring research partnerships are committed to their engagement plans throughout the duration of the grant.

**Implications for the public**

“Nothing about us without us” – this call from disability communities emphasizes the critical need to transform research systems to support meaningful research partnerships. This environmental scan of spinal cord injury research funding organizations in Canada and the United States reveals numerous opportunities for research partnerships. This information can help individuals and organizations to identify funders and programs that meet their research partnership funding needs. Key players in the spinal cord injury research system, including researchers, research users, and funding organizations, should strategically advocate for and consider meaningfully engaging in research partnerships to ensure that diverse perspectives are authentically engaged and valued.

Research discoveries are often not mobilized into practice effectively.^1^ Establishing research partnerships has been identified as one way to help close the research-practice gap.^2-5^ Various definitions and approaches exist for conducting research in partnerships. For example, the Canadian Institutes of Health Research recognizes the value of collaborative research activities between researchers and research users and defines this approach as integrated knowledge translation.^6,7^

 Support for research partnerships is of particular importance to those living with and affected by spinal cord injuries because these individuals often lack access to research discoveries and their priorities are not reflected in the research agenda.^8,9^ The Integrated Knowledge Translation Guiding Principles were developed to support a shift in the way spinal cord injury research is conducted to one that prioritizes “researchers and research users meaningfully working together throughout the research process” (https://ikt.ok.ubc.ca/).^3^ The principles include (1) partners develop and maintain relationships based on trust, respect, dignity, and transparency, (2) partners share in decision-making; see all eight principles in Supplementary file 1. However, these principles will only be as effective as their uptake by individuals and organizations within the spinal cord injury research system, including researchers, research users, research funding organizations, and institutions.^3^ A comprehensive understanding of the approaches to funding and conducting research in partnership can inform strategic efforts to transform research systems for meaningful engagement across spinal cord injury research domains (ie, health research, social sciences and humanities, and natural science and engineering research).^10^

 Research funders play a key role in the spinal cord injury research system because they are organizations and entities that provides financial support needed for research activities.^11^ Research funders can encourage approaches to research, select applications, provide and allocate resources, and evaluate research outputs.^12^ Due to their mandates and power associated with decision-making in distribution of funds, research funders are ideally placed to advance, facilitate and transform systems for meaningful research partnerships.^13^ Research funders have been called to move away from the traditional “fund and forget” model and become active in all forms of knowledge mobilization including in their funding priorities, evaluation criteria, andresearch practices.^13-16^ Funders’ roles in relation to fostering the implementation of research findings have also been highlighted, including advocating for implementation work, monitoring implementation outcomes, and disseminating research findings.^13,14,17^

 Encouragingly, over the past decade several research funding organizations have been recognizing their critical role in supporting all phases of knowledge mobilization (including partnership).^18,19^ However, we still lack information on funders’ positions in the spinal cord injury research system, particularly in their approaches to research partnerships (ie, how they require, evaluate, support research partnerships). Additionally, because of the variations in how researcher funders describe collaborative research and practice,^20^ it remains unclear how research partnerships are being conceptualized by research funders. Therefore, the purpose of this study was to identify major funders of spinal cord injury research in Canada and the United States and identify their approaches to defining, requiring, evaluating, and supporting research partnerships across spinal cord injury disciplines and domains and explore organizational perspectives of the Integrated Knowledge Translation Guiding Principles.

## Methods

###  Design

 We conducted an environmental scan methodology^21^ to gather relevant information about research funding organizations and their approaches to supporting research partnerships.

###  Context and Positionality

 Our research was conducted within the Spinal Cord Injury Integrated Knowledge Translation (SCI IKT) Guiding Principles Partnership. The partnership included funding agency staff members, academic researchers specialized in disability and partnered research, decision-makers in spinal cord injury organizations, and individuals with lived and living experience of spinal cord injury. A sub team of partners co-led this project (named authors) and shared in decision-making at all stages of the research. Table S1 described partners’ positionality and Table S2 outlined their engagement (See Supplementary file 2).

 We were situated within a pragmatic paradigm.^22^ This paradigm was focused on selecting the most feasible and effective methodologies to address research questions and “real-world” issues in the spinal cord injury research system.^23^

###  Procedures

 Our procedures were consistent with the environmental scans conducted for similar research objectives, while allowing flexibility to prioritize funders and programs that aligned with the interests of our research partnership.^24,25^

####  Step 1: Identify Research Funders

 A scientific research database search was conducted in April 2022 to identify research funding organizations in the spinal cord injury research system in the previous five years. The first author (ZS) consulted a university librarian prior to the search. Peer-reviewed spinal cord injury research publications between 2017 and 2022 in Canada and the United States were identified from Web of Science and Scopus because both databases provided a summary of funding sources across the searched publication results. The search strategies were based on recommendations for conducting systematic reviews in spinal cord injury (Supplementary file 2).^26^ Key words including spinal cord injury, paraplegia, and tetraplegia were searched in both databases.

 From each database, the 20 research funding organizations that funded the greatest number of research publications were selected. The results were then compared between the two databases, and only the organizations identified in both databases were retained. We consulted with our partnership team on the comprehensiveness (ie, funders that were missing) and priority (ie, funders that should be prioritized for analysis). The partnership also named funders to be added according to our specific interest in spinal cord injury research and the Canadian context (where our team is situated).

####  Step 2: Identify Funding Programs 

 The organizations’ websites were reviewed to identify programs related to research partnerships. Two researchers (ZS and AS) separately searched for and reviewed keywords (eg, partnership(s), partnered research, integrated knowledge translation, knowledge/research user engagement) on the websites, including program descriptions, announcements, and application guidelines, and identified at least one program relevant to research partnerships. The results were then compared by two researchers. To ensure a feasible number of funders in the scan, the researchers selected the program with the greatest amount per grant when multiple programs were identified for a funder (eg, A program offered an amount of $200 000 per grant and B program offered an amount of $1 000 000 per grant to the awardee. B was selected). In addition, the partnership named the programs that were relevant to the study’s focus in spinal cord injury research partnership (eg, The US Department of Defense’s Spinal Cord Injury Research Program: Clinical Trial Award). Non-research operating funding opportunities, such as service funds, scholarships, and prizes were excluded.

####  Step 3: Extract Information

 Between May 2022 and September 2024, information about selected funding programs was extracted from the websites and online documents, such as application guidelines. Name, objective(s), annual budget of the funding program, grant amounts, grant term, application timeline, review process, and contact for each program were recorded in a data spreadsheet. Information about how the programs define, require, evaluate, support research partnerships was documented. An additional data collection was conducted in July 2025 to address peer-reviewed feedback on inclusion of the National Institutes of Health funding program.

####  Step 4: Interview Informants 

 Informants from the identified funding programs were contacted by email (contact was found on websites) and invited to participate in a videoconference interview with the first or last author (ZS and HG). Eligible participants were (1) affiliated with an institution or organization that funds research partnerships; and (2) residing in Canada or the United States. An interview guide (Supplementary file 2) was developed by our partners and pilot tested with a co-author (GS) who worked for a research funding agency. The interview questions included how the funder defined, required, evaluated, supported research partnerships, as well as their perceptions on the Integrated Knowledge Translation Guiding Principles. The interview guide included questions specific to the individual programs to validate the information extracted online (eg, *What are the overall annual budget, granted amount, and duration of the program?*) and broader questions about the funding organizations (eg, *How does your funding organization define partnered research?*). The procedures were approved by the university research ethics board. All participants gave verbal consent prior to the interviews and were promised that their confidentiality/anonymity was protected.

####  Step 5: Analysis

 Information about each research funder and funding program extracted online was summarized in tables. The categories of information included name, objective(s), annual budget, grant amounts, grant term, application timeline, and review process of each program, as well as how funders define, require, support, evaluate research partnerships. Descriptive statistics were used when applicable (eg, percentage of programs that defined partnership as Integrated Knowledge Translation). Interview data were analyzed using an inductive content approach.^27^ Two co-authors (ZS and AS) reviewed the transcripts and constructed descriptive categories that pragmatically addressed the research questions. The co-authors then discussed their own analysis and consolidated their thoughts into overarching themes. Another co-author (HG) reviewed and provided feedback on the analysis results as a critical friend.^28^ The entire research team reviewed, discussed, and finalized the descriptive themes.

## Results

###  Identified Research Funders

 A total of 25 109 research publications were identified by Web of Science and 3 128 396 were identified by Scopus. Using the funding source summary generated from the two databases, 15 funding organizations were identified from both databases. The National Institutes of Health and its nine affiliated funders were combined as one funder. Next, our partnership added ten funders to the list according to our specific interest in spinal cord injury research and the Canadian context. As a result, the final list included 16 funding organizations (ie, 15 identified from the database search - 9 National Institutes of Health’s affiliated organizations + 10 added by the partnership = 16; Figure).

**Figure:**
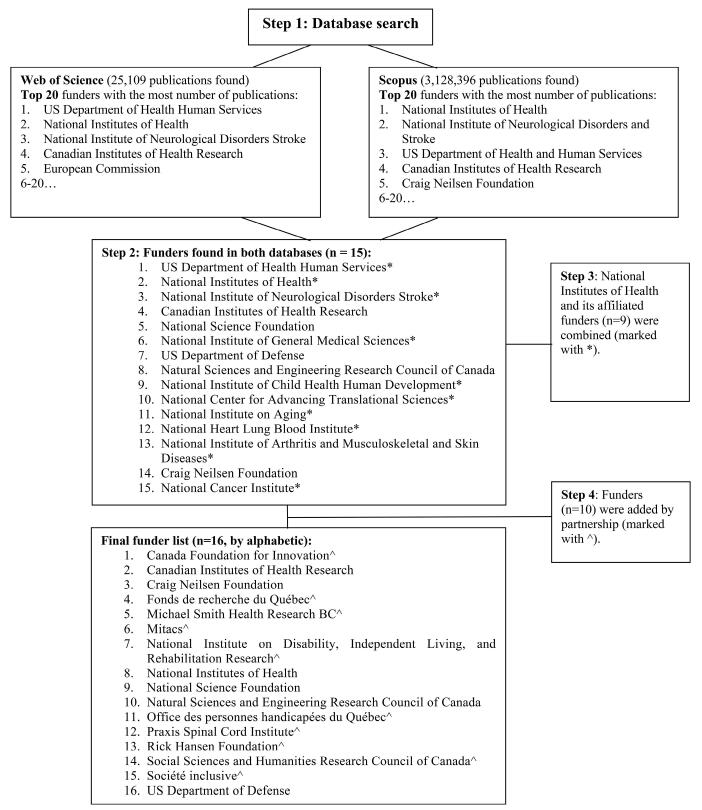
Identified Spinal Cord Injury Research Funders and Partnership Programs.

###  Identified Funding Programs

 At least one research funding program supporting research partnerships was identified for each funder. Two programs were identified for the Michael Smith Health Research BC because the two programs offer the same grant amount (in this case, both were retained because of the inclusion criteria).

####  Program Characteristics

Supplementary file 3 presented the general information of each program identified, including name, objective(s), annual budget, grant amounts, grant term, application timeline, and review process. Eleven of the 17 programs (65%) had a primary objective of supporting research partnerships. Five programs had a specific focus on spinal cord injury. Information on an overall annual budget was only found for seven programs, ranging from $200 000 Canadian dollars (Seed Grant) to $910 000 000 Canadian dollars (Accelerate Program). Individual grant amount ranged from $75 000 Canadian dollars (Accelerate Program) to $3.1 million US dollars (Spinal Cord Injury Research Program: Clinical Trial Award)^[1]^. Individual grant term ranged from 1 to 7 years. Most programs set the application deadline at least 4 to 12 weeks after the announcement. Two programs received applications any time of the year. Thirteen programs adopted a multiple-step review process. Supplementary files 3, 4, and 5 included the information related to the partnership aspect of each program, including definitions, requirements, evaluation, and support research partnerships.

####  Definition

 Despite the common use of words such as “partnership,” “collaboration,” and “co-develop,” the definitions of partnership varied across the funding programs or were not explicitly provided. Seven of the 17 (41%) programs expected partnerships between academic researchers and external organizations, including private, industrial, charitable, community-based, and/or not-for-profit organizations. Six (35%) programs defined partnership as researchers and research users engaging throughout the research process or referred to the definition provided by the Integrated Knowledge Translation Guiding Principles.

####  Requirement

 Eleven (65%) programs required applicants to describe their partnerships in the applications, including the need of partners, the proposed roles, responsibilities, contributions of partners, the activities that will be carried out by partners, and anticipated research benefits for partners. Most of the programs expected partners to actively participate in the research process, while 6 (35%) programs specifically required partners to have in-kind and/or cash contributions. Furthermore, 4 (24%) programs required applications to include one or multiple partner individual(s)/organization(s) as co-lead/co-principal investigators. Six (35%) programs required the involvement of knowledge users (ie, individuals that will use or benefit from the research), including three programs that require the engagement of people with lived and living experience specifically, including both spinal cord injury-specific funding programs (eg, Consumer Program) and generic ones (eg, Strategy for Patient-Oriented Research).

####  Application Evaluation

 Eleven (65%) programs stated their peer review evaluation criteria for proposed partnerships, with the scoring the partnership component varying in its proportional contribution to the overall evaluation. Common peer review criteria included the capacity and expertise of partnerships (particularly in building and maintaining productive partnerships), the extent to which partners engage in and contribute to the research, and the relevancy of potential research outcomes to partners’ needs.

####  Support

 The programs had numerous approaches to supporting research partnerships. Specifically, 6 (35%) programs allowed for remuneration for partners’ engagement and/or cost of collaborative activities (eg, travelling, tele-conferencing) as eligible expenses. Three (18%) programs helped create connections between researchers and potential partners and offered various resources to build applicants’ capacity in developing and maintaining partnerships, such as webinars, workshops, and online platforms. Three (18%) programs helped applicants prepare their applications by providing examples, feedback on drafts of the proposal, and/or explicit descriptions on expected partnership approaches. The Partnership Grant of the Social Sciences and Humanities Research Council of Canada provided applications successful in the letter of intention stage with a grant of up to $20 000 to help them prepare a full application. None of the programs allowed partners to accept/hold research funding.

####  Post-grant Evaluation

 To ensure research teams responsible for delivering on their commitments, meeting agreed-upon objectives, and confirming that resources are used effectively, 14 (82%) programs required interim and/or final reports in the post-grant period. However, we identified few strategies to ensure the responsibilities of research partnerships (eg, meaningful engagement of partners). The Michael Smith Health Research BC’s Convening and Collaborating Program and the Reach Program required awardees to report knowledge users’ involvement in the final report. The FRQ Public-Private Partnership Research Chairs program required the research team to have follow-up committee meetings with the industry partners and requested the partners to assess research progress.

###  Interviews

 Seven of the seventeen research funding organizations agreed to participate in an interview. Interviews averaged 57 minutes in length. A demographic table is not presented to avoid identifying participants. Participants were diverse in terms of age (ranging from 35 to 67), gender identity, race, education level, residency, and disabilities.

 Three themes emerged from the inductive content analysis: (1) Varied support for research partnerships; (2) Minimal capacity for partnership evaluation post-grant; and (3) Need for various tools and resources to further support research partnerships.

####  Varied Support for Research Partnerships

 The informants told us about the different supports that they provide for research partnerships, both during the grant application process and then throughout and at the end of the research project. For example, some funders provide trainings and workshops to help research partnerships navigate the application process and proposed partnership:

 “*We have communications classes and networking classes. We have project management training. So, we use third party facilitators for our training. It’s something that we bring into many of the academic institutions. It really is about that up-skilling and ensuring that we’re setting up these [researchers] for their best success when they’re creating these collaborative innovation projects”* (Funder 3).

 Other research funders provide hands-on support by meeting with teams during the application process and/or throughout the research project:

 “*…with the professional facilitator agent, we are present to help them at any moment of the project because we know that sometimes…there is some tension or need for temporary help and we come and help them at those moments. It’s not [the name of the funder] that is doing the research, it is the research teams, but we are in the background, we are in the landscape of the project”* (Funder 6).

 One agency discussed how it adjusts its approach to supporting research partnerships according to the needs of each partnership:

 “*…once they are awarded the grants, we’ll set up an initial meeting bringing in folks from a few different departments within the organization who might be relevant to the discussion and just basically walk through their grant application and their research protocol. Figure out what they’re planning to do and who they’re planning to do it with. Through those discussions we will identify what their needs are and have subsequent follow-up calls with them, whether that may be quarterly or more or less frequently dependent on their needs. Usually that tends to be a bit front loaded. You know a lot of these conversations happen, usually in the first six months, but there will be periodic follow up as the cycle of the grant goes along”* (Funder 2).

 Further, each organization talked about areas for improvement (eg, follow-up, partnership engagement measurement, etc). One interview captured the movement for more research partnership support that is happening in research:

 “*It is all part of the big culture change towards supporting researchers to work closer with their users and what would that look like. That is a global culture change that needs to be done”* (Funder 1).

####  Minimal Capacity for Tracking and Evaluation Post-grant

 All the agencies reported that holding research partnerships accountable to their plans and meaningful engagement is challenging. When asked if helping research partnerships adhere to their plan and work together meaningfully, one participant explained that there was a lack or capacity to support at this time:

 “*It is relevant, absolutely, and I wouldn’t say it’s beyond our scope. It’s just you know you’ve only got so much time and so many people to do things. If we had, you know, the time and unlimited capacity. Absolutely I would love to try to create something like that but as of yet, we just don’t have the capacity” *(Funder 2).

 Many informants explained that once the funds are delivered to the partnership, their main role in supporting the partnership is over, the rest falls on the partnership. For example, this participant explained that meaningful and ethical engagement relies on the researchers and their academic integrity:

 “*No, that really relies on academic integrity of the research team that we would expect them to honor what they laid out in their research proposal. Once we’ve administered the funds, our involvement is quite limited until an exit report and those exit reports are something that one of our internal teams looks at … Also, as a funder we do put that onus on the academic institution itself as well … There are mechanisms in place within the university and in terms of internal controls to ensure that research is being done in a good way. That’s really all that we can rely on and that comes back to capacity. We are limited in what we can do. To operationalize the oversight of thousands of projects would be hard, that would be really hard” *(Funder 3).

 Further, there remains a lack of understanding of what quality research partnerships look like and how they vary among approaches. This participant summarized the questions that are currently driving work to better understand how to support meaningful research partnership:

 “*So that’s the other part of what we’re trying to do: build capacity and understanding of what partnered research looks like. What is Integrated Knowledge Translation? What is participatory in community-based research and where could those potentially fit, even at the bench [research level]? What would that look like? But we’ve got a long way to go as a world. Everybody’s struggling to help people understand the benefit of this type of work and then what does it actually look like in terms of appropriateness for an individual project?” *(Funder 1).

 With the lack of clarity around how to support meaningful research partnership, funders reported a need for standards and tools to evaluate research partnerships in the peer review and monitor partners’ engagement:

 “*With that, that sort of regular engagement of these research partners, we speak to them, we talk to them, and you know we have relationships with these folks. So, if there’s something that’s not working well, then these things are usually brought to our attention. We do our best to try to help with course correction and improve the actual meaningful and effective engagement. But in terms of, you know, a true measure, we haven’t got something that we can say that this was scoring at 68% and that was scoring at 93%. We just don’t have that” *(Funder 2).

 Despite the lack of clarity around what meaningful research partnership looks like and how to measure it, research funders did discuss the flexibility that research partnerships require. They emphasized that what is meaningful for one partnership, may not be meaningful or helpful for another:

 “*Yeah, I don’t know that being prescriptive on a checklist for how their approach is, is actually helpful in any way. You need to modify your approach depending on who you’re working with. For example, Indigenous communities would be a very different approach than you might use for disabled communities versus rural communities. It is just going to differ all the time. There might be high level principles that you would want to see but specific approaches have to adapt, and they may adapt during the course of the study as well, depending on circumstances that come out”* (Funder 1).

####  Need for Various Tools and Resources to Further Support Research Partnerships 

 All seven interviewed funders commented on the Integrated Knowledge Translation Guiding Principles. Although the funders believed that the principles could serve as a tool in helping to understand and support research partnerships, they reported a need for additional tools and resources:

 “*I think that they are foundational documents to engagement, and I think that they should be utilized by every funder and by every grant applicant and recipient. You know, I think that they create a framework for what meaningful engagement is. It’s like a great tool to check in and make sure that the engagement you’re undertaking is being done with the best intention. There are some pieces that we’re still working to develop, which I think are needed. I think that the guiding principles alone don’t solve the problem of tokenism or unintentional tokenism, whether intentional or unintentional”* (Funder 2).

## Discussion

 This environmental scan identified funders of spinal cord injury research in Canada and the United States and their programs that fund research partnerships. These programs promoted opportunities and resources for research partnerships. Although the programs shared a core value of facilitating research being done in partnership, the research funders differed in how they define, require, evaluate, support research partnerships through their programs. The summary of current approaches provided an initial landscape of funders’ role in the spinal cord injury research system. Furthermore, the results may inform strategic efforts to optimizing meaningful engagement in spinal cord injury research and in a broader context.

 The various ways of how research funders defined partnerships and different types of partners reflect differences in conflation, aggregation of terms, concepts, and frameworks in describing collaborative research and practice.^29^ A lack of definitions may be a reason for funders’ conflation of terms in their application requirements, which makes it challenging for applicants to describe their projects as well as peer reviewers to evaluate those applications.^30^ More explicit terms and definitions for collaborative research and practice are needed. For example, the Patient-Centered Outcomes Research Institute in the United States^31^ and Strategy for Patient-Oriented Research in Canada^32^ both focus on research partnerships that involve patients, their caregivers, and families.

 Further, given the various definitions of research partnerships, it makes sense that research funders have different expectations and requirements. Some research funders require partners to be named as co-lead investigators or to provide in-kind/cash contributions. However, such requirements may not effectively mitigate tokenistic partnerships.^3^ Research funders may consider how to encourage research teams to provide a detailed description of partnership approaches, principles, and/or strategies to help determine if an application aligns with their expectations. For example, the Craig H. Neilsen Foundation requires applicants to demonstrate how their actions align with the Integrated Knowledge Translation Guiding Principles. Research funders can also invite partners to be involved in writing the application or providing letters of endorsement so that partners’ perspectives can be integrated into the conceptualization and planning of the project.

 Varied definitions and requirements for partnership also contribute to varied peer review evaluation of proposed partnerships. Evaluation frameworks, such as the Research Quality Plus for Co-Production,^33^ have been developed to evaluate the quality of co-production. Using such systematic evaluation frameworks can help funders to understand and support research partnerships to deliver on their proposed benefits.

 Despite the increasing call for research partnerships, a general lack of support (eg, time, money, training) for research partnerships from within academia has been identified in the literature.^34^ To help address some barriers experienced by partnerships, our study outlined a range of resources offered by the funding organizations. Specifically, resources that help partnerships navigate their applications and partnered approaches may be valuable for those without prior work experience with the partners, particularly in the initiation stage of a project.^35^ For example, the multiple-stage approach used by the Partnership Grant of the Social Sciences and Humanities Research Council of Canada provided applicants with financial support to their full application. In the implementing and closing stages, allowing the expense of collaborative activities (eg, travelling) can lead to in-person meetings for partnerships to establish and maintain communications, which could be more effective than online meetings.^35^ Moreover, promoting remuneration for partners’ engagement should be considered by more research funders.^36^

 Finally, our results echoed the previous research by highlighting a need for greater capacity for post-grant evaluation from the funders side of research.^10,34^ The funder informants discussed the flexibility that research partnerships require. They emphasized that what is meaningful for one partnership, may not be meaningful or helpful for another. This need for capacity building to support diverse partnerships, ensure partnership responsibilities, and identify what works and what does not (ie, evaluation) aligns with calls from Integrated Knowledge Translation research agendas for principled partnership approaches, transparent reporting, and identified processes, outcomes, and impacts of research partnership.^10^

###  Strengths and Limitations

 First, we may not have captured all relevant North American funders and programs, as our selection was guided by decisions made by the research team. For example, we selected only one funding program per funder to ensure the feasibility of the data extraction and interviews. As a result, some programs were focused on research partnerships more broadly, rather than specifically on spinal cord injury. Although we validated the selection with our partnership team and the interview informants, missing additional partnership-focused programs is still possible. We also recognized that recent shifts in political climate in the United States may have influenced certain aspects of funding programs, such as the use of terms associated with diversity, equity, and inclusion. The value of our study lies in its provision of a point-in-time analysis of funders’ increasing recognition of and support for research partnerships and engagement. Searching results from two databases ensured a broader coverage of various disciplines, while including grey literature could have further expanded the scope of the search. Second, a large part of the results was drawn from these data extracted from the research funders’ websites and online documents. The funders might have other approaches to research partnerships which were not publicly accessible and thus were not captured in this study. Interviews with the research funders supplemented the data. The fact that some funders declined or did not respond to our interview request could have resulted in the provision of selective information about funders in the sample. Last, we acknowledged that we did not collect data on effectiveness and that the information captured (eg, how a funder supports partnership) may not represent the promising practices across partnerships and disciplines. Our intention was to provide an initial landscape of funders’ role in the spinal cord injury research system and to advocate for meaningful engagement in a broader context.

## Conclusions

 To better understand spinal cord injury research funders’ approaches to research partnerships, this environmental scan study was conducted by our SCI IKT Guiding Principles Partnership. Sixteen research funders in Canada and the United States, and seventeen partnership-supportive funding programs were selected and analysed. The results demonstrated that differences exist in how these funders defined, required, evaluated, supported research partnerships through their funding programs. The results provided an initial landscape of funders’ role in the spinal cord injury research system and may inform strategic efforts to optimizing meaningful engagement in spinal cord injury research and in a broader context.

## Acknowledgements

 We would like to thank members of the SCI IKT Guiding Principles Partnership for their contributions and review of this work (See http://www.iktprinciples.com/). We would also like to thank the participants who took the time to participate in this work. Finally, we would like to respectfully acknowledge that we work, and live on traditional and unceded Indigenous territories as well as Treaty lands across Turtle Island which have long served as sites of meeting and exchange amongst nations.

## Members of the SCI IKT Guiding Principles Partnership Panel

 The SCI IKT Guiding Principles Partnership Panel consists of the following members, displayed with their affiliations: Kim Anderson (MetroHealth Medical Center, North American Spinal Cord Injury Consortium, Case Western Reserve University, Cleveland, OH, USA), Peter Athanasopoulos (SCI Ontario, Ontario SCI Solutions Alliance, Toronto, ON, Canada), Myron Campbell (The University of British Columbia Okanagan, Kelowna, BC, Canada), John Chernesky (Praxis Research Institute, North American Spinal Cord Injury Consortium, Vancouver, BC, Canada), Teren Clarke (Spinal Cord Injury Alberta, Calgary, AB, Canada), Victoria Claydon (Simon Fraser University, Burnaby, BC, Canada), Susan Forwell (The University of British Columbia, Vancouver, BC, Canada), Heather Gainforth (International Collaboration on Repair Discoveries, The University of British Columbia Okanagan, Kelowna, BC, Canada), Kathleen Ginis Martin (International Collaboration on Repair Discoveries, The University of British Columbia Okanagan, Kelowna, BC, Canada), Ian Graham (Centre for Practice-Changing Research, University of Ottawa, Ottawa, ON, Canada), Femke Hoekstra (International Collaboration on Repair Discoveries, The University of British Columbia Okanagan, Kelowna, BC, Canada), Anita Kaiser (Canadian Spinal Research Organization, University of Toronto, Toronto, ON, Canada), Anita Kothari (Western University, London, ON, Canada), Amy Latimer-Cheung (Queen’s University, Kingston, ON, Canada), Jocelyn Maffin (SCI British Columbia, Vancouver, BC, Canada), Christopher McBride (SCI British Columbia, Vancouver, BC, Canada), Lowell McPhail (International Collaboration on Repair Discoveries, The University of British Columbia, Vancouver, BC, Canada), Rhyann McKay (University of Alberta, Edmonton, AB, Canada), W. Ben Mortenson (International Collaboration on Repair Discoveries, The University of British Columbia, Vancouver, BC, Canada), Barry Munro (North American Spinal Cord Injury Consortium, Toronto, ON, Canada), Vanessa Noonan (Praxis Research Institute, Vancouver, BC, Canada), Katrina Plamondon (The University of British Columbia Okanagan, Kelowna, BC, Canada), Gayle Scarrow (Michael Smith Health Research BC, Vancouver, Canada), Kimberly Monden (University of Minnesota, Minneapolis, MN, USA), Jasmin Ma (The University of British Columbia, Vancouver, BC, Canada).

## Disclosure of artificial intelligence (AI) use

 Not applicable.

## Ethical issues

 This study is approved by the research ethics board of the University of British Columbia Okanagan [H20-03065].

## Conflicts of interest

 Authors declare that they have no conflicts of interest.

## Endnotes


^[1]^ Most US research funding programs cover principal investigator salary; most Canadian research funding programs do not cover principal investigator salary.

## Supplementary files


Supplementary file 1. The Integrated Knowledge Translation Guiding Principles.

Supplementary file 2 contains Table S1, Table S2, Search Strategies, and Interview Guide.

Supplementary file 3. General Program Information.

Supplementary file 4. Program Information on Partnership Definition and Requirement.

Supplementary file 5. Program Information on Partnership Evaluation, Support, and Post-grant Accountability.
