# Supplementary material for: Funding Programs Relevant to Spinal Cord Injury Research and Their Approaches to Research Partnerships: An Environmental Scan
Source: Int J Health Policy Manag. 2026 Apr 11;15:8813. doi: 10.34172/ijhpm.8813 (PMC13338737; doi:10.34172/ijhpm.8813)
Supplement: Supplementary file 1 — The Integrated Knowledge Translation Guiding Principles. [file ijhpm-15-8813-s001.pdf]

The Integrated Knowledge Translation (IKT) Guiding Principles for Conducting Spinal Cord Injury (SCI) Research in Partnership

What are the IKT Guiding Principles?

The IKT Guiding Principles were systematically co-developed to support IKT partnerships to conduct quality and ethical spinal cord injury (SCI) research that is relevant, useful, useable, and avoids tokenism.

These principles should be used by all partners – researchers, research users, and funders of SCI research – early and throughout the entire research process. Partners should regularly refer to these principles while reflecting on their approach, contributions, and commitment to the partnership and adjust as needed.

Data supporting the effectiveness of the IKT guiding principles are not yet available. For this reason, these are guiding principles and not guidelines.

For definitions and more information go to www.IKTprinciples.com

To engage more meaningfully in research that is relevant, useful, and/or useable, the IKT guiding principles are:

1. Partners develop and maintain relationships based on trust, respect, dignity, and transparency.
2. Partners share in decision-making.
3. Partners foster open, honest, and responsive communication.
4. Partners recognize, value, and share their diverse expertise and knowledge.
5. Partners are flexible and receptive in tailoring the research approach to match the aims and context of the project.
6. Partners can meaningfully benefit by participating in the partnership.
7. Partners address ethical considerations.
8. Partners respect the practical considerations and financial constraints of all partners.

Glossary of Terms

Integrated knowledge translation (IKT): Meaningful engagement of the right research users at the right time throughout the SCI research process.

Meaningful engagement: Contributing to and influencing a personal and socially meaningful research, dissemination, and/or implementation goal and feeling a sense of responsibility to others (e.g. the research team, organizations, people with lived experience of SCI, etc.).

Partners or partnership: Researchers and research users meaningfully engaging throughout the research process.

Principles: Fundamental norms, rules, or beliefs that represent what is desirable and positive for a person, group, organization, or community, and help it in determining the rightfulness or wrongfulness of its actions. Principles are more basic than policy and objectives and are meant to govern both.

Research: The systematic study of a topic in order to understand and/or discover information about the topic.

Research process: Multiple phases in a research project. Phases include but are not limited to planning, conducting, disseminating, and applying the research.

Research users: Individuals or groups that will use or benefit from the research. These groups are different than research participants and include but are not limited to persons with lived experience of SCI, policy-makers, health and/or service providers, other researchers, professional organizations, funders, and industry partners. The partnership should carefully consider who the right research user(s) is/are for its project.

Researcher: A person whose job it is to carry out research activities.

Tokenism: The practice of making only a minimum or symbolic effort to do a particular thing, especially by recruiting a small number of people from under-represented groups in order to give the appearance of equality. Tokenism happens when a partner is asked to endorse, and therefore legitimize, research programs over which they have little real influence or control.

The IKT Guiding Principles were co-developed by a multidisciplinary group of SCI researchers, clinicians, people with SCI, representatives from SCI community organizations, and funding agencies. The IKT Guiding Principles draw on research supported by the Social Sciences and Humanities and Research Council. Want to know more? Visit us at www.IKTprinciples.com
