# Supplementary material for: Funding Programs Relevant to Spinal Cord Injury Research and Their Approaches to Research Partnerships: An Environmental Scan
Source: Int J Health Policy Manag. 2026 Apr 11;15:8813. doi: 10.34172/ijhpm.8813 (PMC13338737; doi:10.34172/ijhpm.8813)
Supplement: Supplementary file 2 — contains Table S1, Table S2, Search Strategies, and Interview Guide. [file ijhpm-15-8813-s002.pdf]

Article title: Funding Programs Relevant to Spinal Cord Injury Research and Their Approaches to Research Partnerships: An Environmental Scan

Journal name: International Journal of Health Policy and Management (IJHPM)

Authors' information: Zhiyang Shi[1], Alanna Shwed[2], Ian D. Graham[3], Gayle Scarrow[4], Peter Athanasopoulos[5], Vanessa K. Noonan[6], John Chernesky[6], Kathryn M. Sibley[7]*, SCI IKT Guiding Principles Partnership Panel#, Heather L. Gainforth[1]*

[1]Department of Kinesiology and Physical Education, McGill University, Montreal, QC, Canada.

[2]School of Health and Exercise Sciences, University of British Columbia Okanagan, Kelowna, BC, Canada.

[3]School of Epidemiology and Public Health, University of Ottawa, Ottawa, ON, Canada.

[4]Michael Smith Health Research BC, Vancouver, BC, Canada.

[5]Spinal Cord Injury Ontario, Toronto, ON, Canada.

[6]Praxis Spinal Cord Institute, Vancouver, BC, Canada.

[7]Department of Community Health Sciences, University of Manitoba, Winnipeg, MB, Canada.

#A full list of the investigators of the IKT Guiding Principles Partnership Panel is provided at the end of the article.

***Correspondence to:** Kathryn M. Sibley; Email: kathryn.sibley@umanitoba.ca & Heather L. Gainforth; heather.gainforth@ubc.ca

Citation: Shi Z, Shwed A, Graham ID, et al. Funding programs relevant to spinal cord injury research and their approaches to research partnerships: an environmental scan. Int J Health Policy Manag. 2026;15:8813. doi:10.34172/ijhpm.8813

Supplementary file 2

Appendix B: Funding agencies in spinal cord injury (SCI) research and their approaches to supporting research partnerships: An environmental scan

Member Name	Organizations	Roles and/or background	Author Information
Zhiyang Shi	University of British Columbia Okanagan<br><br>McGill University	Researcher<br><br>Trainee	ZS is a PhD graduate at McGill University who also works as a research assistant for HG at University of British Columbia Okanagan. ZS identifies as an East-Asian, cis-gender, heterosexual, able-bodied man who has the opportunity to live on the traditional territory of the Kanien'kehà:ka.<br><br>ZS came from China to Canada since 2017 for education. ZS's research focuses on understanding peer support/mentorship for people with SCI and the application of Integrated Knowledge Translation Guiding Principles. ZS's research mainly uses qualitative research methods and partners with community-based organizations.
Alanna Shwed	University of British Columbia Okanagan<br><br>International Collaboration on Repair Discoveries	Researcher<br><br>Trainee	AS is a PhD candidate who works under the supervision of HG at the University of British Columbia Okanagan. AS identifies as a white, cis-gendered, able-bodied woman of settler ancestry living and working on the traditional and unceded territory of the Syilx Okanagan Nation.<br><br>AS's doctoral research aims to support researchers to adopt to IKT Guiding Principles and engage in meaningful SCI research partnerships
Ian D Graham	University of Ottawa and Ottawa Hospital Research Institute	Researcher	IDG is cis-gendered able-bodied man living and working on the ancestral and unceded territory of the Algonquin nation. He is Distinguished Professor in the School of Epidemiology and Public at the University of Ottawa and a Senior Scientist at the Ottawa Hospital Research

			Institute. Agencies. For six years he was seconded to the Canadian Institutes of Health Research to hold the position of Vice President of Knowledge Translation. His research program focuses on implementation science, knowledge translation and integrated knowledge translation. He has also studied health funding agencies and their approaches to KT and IKT.
Gayle Scarrow	Michael Smith Health Research BC	Leadership Role in SCI funding organization	GS is the director, knowledge translation at Health Research BC. GS is a white, cis-gender, heterosexual woman of settler ancestry living and working on the traditional and unceded territory of the xʷməθkwəy̓əm (Musqueam), Səl̓ílwətaʔ/Selilwitulh (Tsleil-Waututh) and Skwxwú7mesh (Squamish) Peoples in Vancouver, British Columbia, Canada. Her work aims to foster and accelerate the impact of health research in BC and beyond.
Peter Athanasopoulos	SCI Ontario	Leadership Role in SCI funding organization<br><br>Person with lived experience of SCI	Not provided
Vanessa Noonan	Praxis Spinal Cord Institute	Leadership Role in SCI funding organization<br>Researcher	VN is a white, cis-gender, straight, able-bodied woman of settler ancestry living and working on the traditional and unceded territory of the Squamish, Musqueam and Tsleil-Waututh First Nations. VN grew up in Vancouver, BC, Canada and is of English descent.<br><br>She works as the Director of Research and Best Practice Implementation at the Praxis Spinal Cord Institute. In her role, she oversees the Cure and Care Programs, where the aim is to translate knowledge into action.
John Chernesky	Praxis Spinal Cord Institute	Leadership Role in SCI funding organization	Not provided

		Person with lived experience of SCI	
Kathryn Sibley	University of Manitoba	Researcher	KS is a white, cis-gender, heterosexual, neuro-typical woman. KS grew up in Oakville, Ontario, Canada and am of English, Scottish, and Irish settler descent. KS has a BSc in Kinesiology from the University of Waterloo and an MSc in rehabilitation sciences and PhD in medical sciences from the University of Toronto. KS completed postdoctoral training at the Toronto Rehabilitation Institute before relocating to Winnipeg, Manitoba, Canada where she has lived since 2014. KS is an Associate Professor in the Department of Community Health Sciences, Max Rady College of Medicine, with a cross-appointment in the College of Rehabilitation Sciences, at the University of Manitoba in Winnipeg, Canada, Treaty 1 territory and the homeland of the Red River Metis nation. KS holds a Canada Research Chair in integrated knowledge translation in rehabilitation sciences. KS serves as Director of knowledge translation at the Centre for Healthcare Innovation, home of Manitoba's CIHR SPOR (Strategy for Patient-Oriented Research) SUPPORT Unit.<br><br>She studies and attempts to improve the integration of health research and care through knowledge translation theories and research methods in partnership with knowledge users. She has had a longstanding focus on fall prevention in older adults, and more recently has been learning about intersectionality and considering how to more explicitly advance equity and diversity through knowledge translation.

SCI IKT Guiding Principles Partnership Panel	N/A	N/A	N/A
Heather L. Gainforth	University of British Columbia Okanagan<br><br>International Collaboration on Repair Discoveries	Researcher	HG is an Associate Professor at the University of British Columbia and an ICORD Principal Investigator. HG is a white, cis-gender, straight, able-bodied woman of settler ancestry living and working on the traditional and unceded territory of the Syilx Okanagan Nation.<br><br>She researches in the areas of Behaviour Change and Integrated Knowledge Translation. Her research aims to combat tokenism in science and foster meaningful engagement between researchers and research users by advancing the science and practice of integrated knowledge translation.

Notes. SCI = Spinal Cord Injury

Appendix C: Partner Engagement

The table below outlines the involvement of partner members through the research process. The table includes an overview of the research activities that took place and which partner members were involved.

**Research Activity**	**Partner Members Involved**	**Activity Description**
Conceptual Design – Grant Application	HG and the SCI Guiding Principles Panel	HG and the SCI Guiding Principles Panel submit a SSHRC Partnership Development Grant that includes a study to understand SCI research funding agencies' approaches to supporting research partnerships.<br><br>The grant was co-written and reviewed by all team members.
Conceptual Design – Finalizing Study Materials and Methods	ZS, HG, AS, IG, GS, PA, VN, JC, KS	ZS, HG, and AS planned and hosted a meeting with all co-authors to discuss the study design.<br><br>The meeting agenda included having the partnership provide broad feedback on the following study components: data resources; study documents (i.e., interview guides); inclusion criteria & sampling methods; recruitment methods.<br><br>Decisions on study materials and methods were made upon the agreement of all co-authors.
Conceptual Design – Creation and Pilot Test of Interview Guide	ZS, HG, AS, GS	ZS, HG, and AS created the first draft of the interview guide. HG reviewed the interview guide;<br><br>ZS conducted an initial pilot interview with GS. Based on the pilot, GS provided feedback on the interview guide;<br><br>ZS and AS submitted interview guide revisions to UBC Research Ethics Board.
Data Collection – Online Information Extraction	ZS, AS	ZS conducted a database search and identified SCI research funding agencies and programs.<br><br>ZS and AS extracted information on the funding agencies' websites.

		ZS organized the information extracted into multiple tables as preliminary results.
Recruitment	ZS, HG, AS	ZS, HG, and AS sent individualized recruitment e-mails to funding agencies who would be eligible to participate.
Data Collection – Conducting Interviews	ZS	ZS conducted interviews with all interested informants of the funding agencies.
Data Analysis	ZS, HG, AS, IG, GS, PA, VN, JC, KS	All co-authors involved in study design were emailed a copy of the proposed data analyses and methods and discussed the preliminary results in an online meeting.
Data Analysis – Interview Data Analysis	AS, ZS, HG	AS and ZS employed the steps of a content analysis to all interview transcripts;<br><br>HG acted as a critical friend for the interview data results.
Manuscript Preparation	ZS, HG, AS, IG, GS, PA, VN, JC, KS	ZS, HG, AS led the writing of the manuscript. The manuscript was sent to all named co-authors and editorial comments were addressed.<br><br>The edited manuscripts were sent to the broader partnership panel for final approval and feedback prior to submission.<br><br>All named authors approved the final version of the manuscript.

Appendix D: Search Strategies

- Web of Science

#1 ALL=(spinal cord)
#2 ALL=(Spinal Cord Injur*)
#3 (ALL=(spine) OR ALL=(spinal)) AND (ALL=(facture*) OR ALL=(wound*) OR ALL=(trauma*) OR ALL=(injur*) OR ALL=(damag*))
#4 ALL=(paraplegi*)
#5 ALL=(tetraplegi*)
#6 ALL=(quadriplegi*)
#7 ALL=(Spinal Cord Ischemia)
#8 ALL=(central cord injury syndrome)
#9 ALL=(myelopath*) AND (ALL=(traumatic) OR ALL=(post-traumatic) OR ALL=(ischemi*))
#10 ALL=(spinal cord) AND (ALL=(contusion*) AND ALL=(laceration*) OR ALL=(transection*) OR ALL=(ischemi*) OR ALL=(syndrome))

OR #1~10 AND USA or Canada (Countries/Regions) and 2022 or 2021 or 2020 or 2019 or 2018 or 2017 (Publication Years)

- Scopus

#1 TITLE-ABS-KEY=(spinal cord)
#2 TITLE-ABS-KEY=(Spinal Cord Injur*)
#3 TITLE-ABS-KEY=(Spinal or spine) W/1 (fracture* or wound* or injur* or damage*)
#4 TITLE-ABS-KEY=(paraplegi*)
#5 TITLE-ABS-KEY=(tetraplegi*)
#6 TITLE-ABS-KEY=(quadriplegi*)
#7 TITLE-ABS-KEY=(Spinal Cord Ischemia)
#8 TITLE-ABS-KEY=(central cord injury syndrome)
#9 TITLE-ABS-KEY=myelopath* W/1 (traumatic OR post-traumatic OR ischemi*)
#10 TITLE-ABS-KEY{spinal cord} W/1 (contusion* OR laceration* OR transection* OR ischemi* OR syndrome)

OR #1~10 AND (LIMIT-TO (AFFILCOUNTRY , "united states") OR LIMIT-TO (AFFILCOUNTRY , "canada")) AND (LIMIT-TO (PUBYEAR , 2022) OR LIMIT-TO (PUBYEAR , 2021) OR LIMIT-TO (PUBYEAR , 2020) OR LIMIT-TO (PUBYEAR , 2019) OR LIMIT-TO (PUBYEAR , 2018) OR LIMIT-TO (PUBYEAR , 2017))

Appendix E: Interview Guide

Consent Questions:

- Before we move further with the interview, do you have any questions about the interview or consenting to participate?
- *If a zoom call* - Before I hit record, if you want to further protect your privacy you may change your zoom name to a nickname or alternative name and/or turn off your video.
- Do you consent to have your interview recorded?

Section 1: Introduction Questions (5 minutes)

1. Can you tell me about your role in your organization?
2. Can you tell me how long you have been with your organization?

Section 2: Partnered Research Questions (30 minutes)

1. From your website, we found that your organization offers funding program(s) to support research partnerships. [*Program name depending on the organization*] is the program(s) that aims to support partnered research.
    a. Can you tell me more about that program?
    b. What are the overall annual budget, granted amount, and duration of the program?
    c. Is there any other program that also supports partnered research?
2. How does your funding organization define partnered research? / what are you looking for from a research partnership?
3. How does your funding organization require partnered research in the application? / what kind of partnership are you expecting?
4. How does your funding organization support partnered research throughout the application process?
    a. Any budget for knowledge users' engagement?
    b. How do you ensure the accountability (i.e., meaningful engagement) of research partnerships?

Section 3: IKT Guiding Principles Questions (15 minutes)

I have sent you the link to the IKT Guiding Principles over the zoom chat that I also emailed you in the scheduling email. Are you able to open that up for me? We noticed that your program(s) have/have not adopted the IKT Guiding Principles.

1. Have you seen the principles? What do you think about the principles?

2. Why does/does not your Do you have any questions or comments on the IKT Guiding Principles?

3. How do/ do not the principles fit within your organization's current approach to funding research?

4. Why your organization has/has not adopted the principles?

Section 4: Closing questions (5 minutes)

1. Do you have anything you would like to add or any questions that I can address?

Section 5: Demographics and Gift Card (5 minutes)

To end the interview, I have a few demographics questions for publishing purposes. You do not need to answer these questions if you would prefer not to respond.

1. Name of the organization that you work for?

2. What year were you born?

3. What gender do you identify as?

4. What province or state do you reside in?

5. What ethnicity do you identify as?

6. What is the highest level of education you have completed?

7. Do you have a spinal cord injury?
   a. Year of SCI
   b. Tetraplegia or paraplegia?
   c. Injury complete or incomplete?
   d. Primary mode of mobility outside your home?

8. What gift card would you like to receive?
   a. Amazon
   b. Starbucks
   c. Indigo
   d. Bath and Body Work
