# Supplementary material for: Funding Programs Relevant to Spinal Cord Injury Research and Their Approaches to Research Partnerships: An Environmental Scan
Source: Int J Health Policy Manag. 2026 Apr 11;15:8813. doi: 10.34172/ijhpm.8813 (PMC13338737; doi:10.34172/ijhpm.8813)
Supplement: Supplementary file 3 — General Program Information. [file ijhpm-15-8813-s003.pdf]

Article title: Funding Programs Relevant to Spinal Cord Injury Research and Their Approaches to Research Partnerships: An Environmental Scan

Journal name: International Journal of Health Policy and Management (IJHPM)

Authors' information: Zhiyang Shi[1], Alanna Shwed[2], Ian D. Graham[3], Gayle Scarrow[4], Peter Athanasopoulos[5], Vanessa K. Noonan[6], John Chernesky[6], Kathryn M. Sibley[7]*, SCI IKT Guiding Principles Partnership Panel#, Heather L. Gainforth[1]*

[1]Department of Kinesiology and Physical Education, McGill University, Montreal, QC, Canada.

[2]School of Health and Exercise Sciences, University of British Columbia Okanagan, Kelowna, BC, Canada.

[3]School of Epidemiology and Public Health, University of Ottawa, Ottawa, ON, Canada.

[4]Michael Smith Health Research BC, Vancouver, BC, Canada.

[5]Spinal Cord Injury Ontario, Toronto, ON, Canada.

[6]Praxis Spinal Cord Institute, Vancouver, BC, Canada.

[7]Department of Community Health Sciences, University of Manitoba, Winnipeg, MB, Canada.

#A full list of the investigators of the IKT Guiding Principles Partnership Panel is provided at the end of the article.

***Correspondence to:** Kathryn M. Sibley; Email: kathryn.sibley@umanitoba.ca & Heather L. Gainforth; heather.gainforth@ubc.ca

Citation: Shi Z, Shwed A, Graham ID, et al. Funding programs relevant to spinal cord injury research and their approaches to research partnerships: an environmental scan. Int J Health Policy Manag. 2026;15:8813. doi:10.34172/ijhpm.8813

Supplementary file 3. General Program Information

Funding Agency	Funding Program	Objective	Overall Annual Budget	Grant Amount	Grant Term	Application Timeline	Review Process	SCI Specific
Canada Foundation for Innovation	College Fund	To support partnerships between colleges and a range of public, private or not-for-profit partners.	Unknown ($92 million CAD in history)	$60,000 to one million CAD	Varies	Announcement: Unknown<br>Notice of intent: Spring<br>Application deadline: Summer	3-step review:<br>1. Administrative review<br>2. Expert Committees review<br>3. Multidisciplinary Assessment Committee review	No
Canadian Institutes of Health Research	Strategy for Patient-Oriented Research (SPOR)	Enable investigators to co-create and undertake comparative effectiveness and/or implementation science innovative Clinical Trials (iCTs) with patient partners and multidisciplinary teams by supporting the development and testing of innovative methodologies and design, integrating the principles of SPOR.<br>Support the application of iCT designs, including advancements in trauma-informed designs; equity, diversity and inclusion; and inclusion of individuals who have historically been excluded.<br>Build capacity for iCT methods and research through increased intensity, i.e., number of trialists, strengthening iCT research capability nationally, in	$22,000,000 CAD	Up to $2,000,000 CAD	Up to 4 years	Announcement: Winter<br>Letter of intent: Spring<br>Application deadline: Summer	2-step review:<br>1. A relevance review will be conducted at the Letter of Intent (LOI) phase.<br>2. A CIHR review committee will evaluate the full applications. The multi-disciplinary, diverse, international panel will include patient engagement experts, patients, health care professionals, policy makers and researchers with expertise in comparative effectiveness research, implementation science research, Indigenous knowledge/research, patient-oriented research (POR), and iCT.	No

		alignment with the SPOR Capacity Development Framework.						
Craig H. Neilsen Foundation	SCI Research on the Translational Spectrum (SCIRTS) Senior Research Grants	Supporting the wide array of research possibilities and opportunities, with the Spinal Cord Injury Research on the Translational Spectrum (SCIRTS) portfolio, we seek to improve the understanding of traumatic spinal cord injury and develop new approaches to alleviate the dysfunction and complications that follow.	Unknown	Up to $800,000 USD (with an annual budget under $300,000)	Up to 3 years	6 weeks between announcement and letter of intent deadline (Fall)	Peer-reviewed by members of the Neilsen Foundation's Review Board.	Yes
Fonds de recherche du Québec	FRQ Public-Private Partnership Research Chairs program	To support sustainable partnerships between private companies and Québec universities by developing research capacity and supporting a new generation of researchers in order to generate significant economic benefits for Québec.	$4,000,000 CAD	Up to $200,000 CAD per year	3 to 5 years	Announcement : Unknown<br>Notice of intent: Fall<br>Application deadline: Fall	2-Step review:<br>1. Scientific assessment by an evaluation committee (EC) composed of peers and representatives from various public and private research organizations at both the national and international levels.<br>2. Evaluation on the basis of the added value of the research program for Québec's economic recovery. This evaluation will be carried out by a multidisciplinary evaluation committee (MEC) whose composition will be representative of the Fonds and the research areas of the applications	No

Michael Smith Health Research BC	Convening & Collaborating Program (C2)	To bring together researchers and research users to co-develop research to increase the likelihood that the research findings will be relevant to users.	Unknown	Up to $15,000 CAD	18 months (projects/activities must be planned to be completed within 12 months)	Announcement: Spring<br>Applicant deadline: Spring<br>Host institution deadline: Spring	2-Step review:<br>1. Eligibility screen by internal staff<br>2. External review panel consisting of researchers and KT specialists	No
Michael Smith Health Research BC	Reach Program	To bring together researchers and research users to support the dissemination of research outputs, within the context of an established body of knowledge, in ways that facilitate its effective uptake and potential to impact health and care.	Unknown	Same as C2	Same as C2	Same as C2	Same as C2	No
Mitacs	Accelerate Program	To build research partnerships while funding students and postdocs.	$910,000,000 CAD	Varies, starting at $7,500 CAD	4 months to 5 years	Apply any time	3-Step review:<br>1. Completion and eligibility screen<br>2. Internal review by research team with diverse expertise<br>3. External review by 3 academic members	No
National Institute on Disability, Independent Living and Rehabilitation Research (U.S.)	Spinal Cord Injury Model System Multi-Site Collaborative Research Project	The purpose of this SCIMS multi-site collaborative research project is to utilize the collaborative capacity of the Spinal Cord Injury Model System (SCIMS) Centers to conduct high quality multi-site research toward improving outcomes	Unknown	$895,000 to $900,000 USD	5 years	Announcement: Spring<br>Letter of intent: Spring<br>Application deadline: Spring	3-Step review:<br>1. Eligibility screen.<br>2. Independent review panel of at least 3 individuals<br>3. Final decisions made by ACL administrators	Yes

		of people with SCI.						
National Institutes of Health (U.S.)	NIH Research Project Grant	To support a discrete, specified, circumscribed project in areas representing the specific interests and competencies of the investigator(s).	Unknown	Not limited	Up to 5 years	Announcement : Winter<br><br>Application deadline: Multiple deadlines between February and October	2-Step review:<br>1. Scientific Merit Review<br>2. Advisory Council Review	No
National Science Foundation (U.S.)	Partnerships for Innovation – Research Partnership (PFT-RP) track	To translate prior NSF-funded research results in any field of science or engineering into technological innovations with promising commercial potential and societal impact by supporting instead complex, multifaceted technology development projects that are typically beyond the scope of a single researcher or institution and require a multiorganizational, interdisciplinary, synergistic collaboration.	Unknown	Up to $1,000,000 USD	36 months	Application opens three times a year in Spring, Fall, and Winter<br><br>Application deadline: Unknown	2-Step review:<br>3. Ad hoc Review<br>4. Panel Review	No
Natural Sciences and Engineering Research Council of Canada	Alliance Grant	Alliance grants encourage university researchers to collaborate with partner organizations, which can be from the private, public or not-for-profit sectors. These grants support research projects led by strong,	Unknown	$20,000 to one million CAD per year	1 to 5 years	Apply any time	Depending on the scale of the projects. In general, applications are reviewed by external reviewers with expertise directly related to the proposal. Review time range from 5 to 24 weeks.	No

		complementary, collaborative teams that will generate new knowledge and accelerate the application of research results to create benefits for Canada.						
Office des personnes handicapées du Québec	Programme de subventions à l'expérimentation	To financially support experimental projects, studies, and research to increase the social participation of people with disabilities.	Unknown	Up to $100,000 CAD	Up to 24 months	Announcement: unknown<br>Application deadline: Spring	2-step review:<br>1. Eligibility screen<br>2. Review by evaluation committee made up of advisors and recognized external experts in the field covered by the project	No
PRAXIS Spinal Cord Institute	Consumer Program	To increase consumer involvement in a multi-disciplinary collaborative partnership between researchers, care providers and industry for maximum impact on health and quality of life of those living with SCI.	$1,000,000 CAD	Varies	Varies	8 to 12 weeks between announcement and application deadline	3-Step review:<br>1. Eligibility screen by internal staff.<br>2. Internal review, including members with lived experience.<br>3. External review, including members with lived experience.	Yes
Rick Hansen Foundation	International Collaboration on Repair Discoveries (ICORD) Seed Grants	To provide seed funding for novel research projects proposed by Principal Investigators and Investigators within ICORD and enable ICORD faculty members to generate pilot data for future applications for multi-year operating grants (especially Tri-council) as a result of receiving a Seed Grant.	$200,000 CAD/year (two annual competitions)	$20,000 CAD	Up to 2 years	4 to 6 weeks between announcement and application deadline	One-step review by an adjudication committee consisting of ICORD managing director and 3 or more previous awardees.	Yes

Social Sciences and Humanities Research Council of Canada	Partnership Grant	To support formal partnerships between academic researchers, businesses and other partners that will advance knowledge and understanding on critical issues of intellectual, social, economic and cultural significance. By fostering mutual co-operation and sharing of intellectual leadership, the grants allow partners to innovate, build institutional capacity and mobilize research knowledge in accessible ways. The grants may also be used to help establish partnered chairs and research centres.	Unknown	Stage 1 (letter of intention): up to $20,000 CAD<br>Stage 2 (formal application): up to $2,500,000 CAD (with an annual budget under $500,000 CAD)	4 to 7 years	Announcement: Unknown<br>Letter of Intention: Winter<br>Application Deadline: Fall	3-step review:<br>1. Letter of intention reviewed by merit review committee<br>2. Formal application reviewed by expert review panels (3~6 experts)<br>3. The multidisciplinary merit review committee reviews the applications and reports from expert reviews.	No
Société inclusive	Inclusive Society Partnership Research Program	The program will support innovative projects that aim to:<br>• Produce concrete changes in the lives of people with disabilities.<br>• Support the transformation of associated ecosystems (caregivers, community organizations, public agencies)<br>• Implement solutions to reduce the environmental and social barriers that people with disabilities	Unknown	Up to $35,000 CAD	12-18 months	Announcement: Fall<br>Submission deadline: Winter	3-Step review:<br>1. Eligibility.<br>2. Review committee of four members, including two researchers, one partner, and one funder representative.<br>3. Review across committees.	No

		face in their daily activities.						
United States Department of Defense	Spinal Cord Injury Research Program: Clinical Trial Award	To fund the rapid implementation of clinical trials with the potential to have a significant impact on the treatment or management of spinal cord injury (SCI).	$19,520,000 USD	Up to 3,100,000 USD	Up to 4 years	Announcement : Spring<br>Preproposal: Spring<br>Application deadline: Fall	2-Step review:<br>1. Peer review (each application is assessed for its own merit, independent of other applications)<br>2. Programmatic review (a comparison-based process in which applications with high scientific and technical merit are further evaluated for programmatic relevance)	Yes

Note: Funders are listed in alphabetic order. Information gathered represent the year of 2021 or 2022.
*Currency exchange rate: 1 USD ≈ 1.4 CAD.
