# Supplementary material for: Funding Programs Relevant to Spinal Cord Injury Research and Their Approaches to Research Partnerships: An Environmental Scan
Source: Int J Health Policy Manag. 2026 Apr 11;15:8813. doi: 10.34172/ijhpm.8813 (PMC13338737; doi:10.34172/ijhpm.8813)
Supplement: Supplementary file 4 — Program Information on Partnership Definition and Requirement. [file ijhpm-15-8813-s004.pdf]

Article title: Funding Programs Relevant to Spinal Cord Injury Research and Their Approaches to Research Partnerships: An Environmental Scan

Journal name: International Journal of Health Policy and Management (IJHPM)

Authors' information: Zhiyang Shi[1], Alanna Shwed[2], Ian D. Graham[3], Gayle Scarrow[4], Peter Athanasopoulos[5], Vanessa K. Noonan[6], John Chernesky[6], Kathryn M. Sibley[7]*, SCI IKT Guiding Principles Partnership Panel#, Heather L. Gainforth[1]*

[1]Department of Kinesiology and Physical Education, McGill University, Montreal, QC, Canada.

[2]School of Health and Exercise Sciences, University of British Columbia Okanagan, Kelowna, BC, Canada.

[3]School of Epidemiology and Public Health, University of Ottawa, Ottawa, ON, Canada.

[4]Michael Smith Health Research BC, Vancouver, BC, Canada.

[5]Spinal Cord Injury Ontario, Toronto, ON, Canada.

[6]Praxis Spinal Cord Institute, Vancouver, BC, Canada.

[7]Department of Community Health Sciences, University of Manitoba, Winnipeg, MB, Canada.

#A full list of the investigators of the IKT Guiding Principles Partnership Panel is provided at the end of the article.

***Correspondence to:** Kathryn M. Sibley; Email: kathryn.sibley@umanitoba.ca & Heather L. Gainforth; heather.gainforth@ubc.ca

Citation: Shi Z, Shwed A, Graham ID, et al. Funding programs relevant to spinal cord injury research and their approaches to research partnerships: an environmental scan. Int J Health Policy Manag. 2026;15:8813. doi:10.34172/ijhpm.8813

Supplementary file 4. Program Information on Partnership Definition and Requirement

Funding Agency	Funding Program	Definition of Partnership	Requirement for Partnership
Canada Foundation for Innovation	College Fund	Partnership between researchers and industry/community.	Applications must include:<br>• What are the needs of your industry or community and how did you determine them (e.g. stakeholder consultations, environmental scan)?<br>• What applied research activities will you undertake with your partners? For each, include the following: What are the objectives? What is the methodology? What are the intended outcomes?<br>• What is your college's track record of establishing and maintaining research partnerships with the industry or community? Who are your partners for the proposed applied research activities?<br>• How did you select your partners?<br>• How will you reach out to the industry or community to stimulate new partnerships?<br>• What are the specific challenges or systemic barriers that exist in the context of your research program that could prevent individuals from underrepresented groups from participating equitably within the research team?<br>• Describe at least one concrete practice that you put in place to overcome the challenges or systemic barriers previously described and which demonstrates that equity and diversity were intentionally considered when composing the research team and recruiting team members.<br>• Describe at least one concrete practice that you will adopt to facilitate the ongoing inclusion of underrepresented groups in the research team, and how you will implement that best practice given the challenges or systemic barriers previously described.<br>• What are the anticipated benefits of the applied research activities for the industry or community beyond the outcomes described in the "Applied research" section? This can include knowledge mobilization and technology transfer activities.
Canadian Institutes of Health Research	Strategy for Patient-Oriented Research (SPOR)	A continuum of research that engage patients, including people with	The core leadership of the research team must include at least five Principal Applicants (PAs) / Principal Knowledge Users (PKUs), covering each of the

		lived/living experience, as partners throughout the research process, focuses on patient-identified priorities and improves patient outcomes. The research, conducted by multidisciplinary teams in partnership with relevant stakeholders, aims to apply the knowledge generated to improve health outcomes for Canadians.	following categories and should aim to represent the diversity of the Canadian population:<br>• A patient (including people with lived experience);<br>• Two researchers, including at least one Early Career Researcher (ECR);<br>• A health care provider (e.g., physician; nurse; occupational therapist; Indigenous Elder, healer or Knowledge Keeper; pharmacist; social worker; physiotherapist; psychologist; or other health professional); and<br>• A health system decision maker (i.e., individual empowered to engage with the research project and serve as a conduit of information to and from their respective health system, health care institution, health care centre [including health or wellness centres on-reserve or in urban centres], etc.).<br>Applicants must secure partner contributions from non-federal sources to match the CIHR contribution at a minimum 1:1 ratio.
Craig H. Neilsen Foundation	SCI Research on the Translational Spectrum (SCIRTS) Senior Research Grants	Support the use of Integrated Knowledge Translation Guiding Principles: Researchers and research users meaningfully engaging throughout the research process.	Including persons with lived experience and/or knowledge translation expertise to research teams is encouraged but not required.
Fonds de recherche du Québec	FRQ Public-Private Partnership Research Chairs program	Partnership between researchers and industry partners/private companies, referred to as industry partners.	The industry partner(s)’ commitment to the C3P includes cash and in-kind contributions:<br>• Minimal of $100,000 (cash) and $15,000 (in-kind)<br>Industry partners participate in the development and implementation of the C3P research program and in the training of HQP. They will also be able to use and exploit the research results produced by the C3P.<br>Applications must include:<br>• A letter from each partner confirming their commitment and contribution to the C3P;<br>• Declaration of Interest from each partner identifying any relationship between them and the principal investigators.
Michael Smith Health Research BC	Convening & Collaborating Program (C2)	Researchers and research users to co-develop research so as to increase the	Applications must include a research user co-lead.

		likelihood that the research findings will be relevant to users.	Research user co-lead needs to provide information on:<br>• Organization affiliations, job position.<br>• Qualifications and Expertise: Describe how your experience and qualifications make you well-suited for your role in co-leading the activities proposed in the application.<br>• What are your relevant skills and experiences?<br>• What KT activities you currently and previously engaged in?<br>Applications must include an IKT plan: a detailed description of the convening and collaborating activities and how these activities are appropriate for the purpose and for the participants.
Michael Smith Health Research BC	Reach Program	Same as C2.	Same as C2.
Mitacs	Accelerate Program	Partnership between academic researchers and not-for-profit organizations in Canada.	Applications must include:<br>• A detailed description of the activities that will be performed on-site at the partner organization and the expected interaction with and supervision by employees of the partner organization.<br>• Indicate the resources the partner organization will be providing to support the intern's work at their premises. Include information about (1) space, (2) resources, and (3) expertise that will be provided by the organization to the intern.<br>• The partner's proposed role in the project.<br>• How the partner will benefit from participating.<br>Partner organizations need to have a financial contribution starting at $7500.
National Institute on Disability, Independent Living and Rehabilitation Research (U.S.)	Spinal Cord Injury Model System Multi-Site Collaborative Research Project	Partnerships between Spinal Cord Injury Model System (SCIMS) centers.	Applicants must be collaborating with 3 or more of the NIDILRR-funded SCIMS centers.<br>Applications must be ensuring that the input of people with SCI is used to shape the SCIMS collaborative research throughout the course of the grant.<br>Applications must include:<br>• A Work Plan. The Work Plan should include a statement of the project's overall goal(s), anticipated outcome(s), and the major tasks that are proposed to achieve

			the goal and outcome(s). For each major task, the Work Plan should identify timeframes involved and the lead person responsible for the task.<br>• Letters of commitment from key participating organizations and agencies.<br>• Summary of involved individuals and organizations.
National Institutes of Health (U.S.)	NIH Research Project Grant	Not found.	Not found.
National Science Foundation (U.S.)	Partnerships for Innovation – Research Partnership (PFT-RP) track	Partnerships between academic researchers and third-party organizations such as industry, non-academic research organizations, federal laboratories, public or non-profit technology transfer organizations, or other universities.	Applications must include:<br>• A co-PI who is a member or employee of the required Industrial Partner organization.<br>• Describe the partnership that is being assembled to pursue the use-inspired research project. Discuss the capabilities of each of the partners and their roles in the project.<br>• How will the proposed partnership achieve the goals of the PFI project to a) catalyze and accelerate technology development toward commercialization, and b) contribute to the educational goals of the program?<br>• Provide an assessment plan that will help gauge the success of the research partnership(s) and third-party collaboration(s). Discuss your choice of the stated success metrics.
Natural Sciences and Engineering Research Council of Canada	Alliance Grant	Partnership between researchers and private, public, or not-for-profit organizations.	Each partner organization must play an active role in the project and make in-kind contributions through at least one of the following roles:<br>• Active participation in project research activities.<br>• Application of project research results to help achieve the desired outcomes.<br>• Active participation in translating or mobilizing the knowledge produced by the project to generate the greatest possible economic, social, and/or environmental benefits for Canada and Canadians.<br>Applications must include:<br>• List all partner organizations expected to play a key role in the activities or to make cash and/or in-kind contributions.<br>• Describe the core activity of the partner organizations and their experience related to the research project, such as any efforts to date that the partner

			organizations have invested toward addressing this problem, the need for this research project, and how the topic is relevant and aligned with the partner organizations' activities.<br>• Explain how each partner organization will be actively involved (through cash and/or in-kind contributions) in co-designing and implementing the research program. Describe the value added through in-kind contributions and how these are important to realizing the project's intended outcomes.<br>• Outline each partner organization's strategy and capacity to translate the research results into practical application to achieve the desired outcomes and impacts, including any planned knowledge translation activities and integration of the research results into its operations.
Office des personnes handicapées du Québec	Programme de subventions à l'expérimentation	Not found	Partnership is encouraged but not required.<br>Application must include collaborative agreements with partners, if applicable.
PRAXIS Spinal Cord Institute	Consumer Program	Consumer engagement is founded on the understanding that those affected by a decision have a right to be a part of the decision-making process and that their input will influence that decision. Engagement occurs when consumers have a meaningful and active role in decision-making, setting priorities, conducting research, and translating research knowledge for the benefit of its intended users.	Applications must include:<br>• People with lived experience.<br>• An engagement plan.
Rick Hansen Foundation	International Collaboration On Repair Discoveries (ICORD) Seed Grants	Not found.	Not found.
Social Sciences and Humanities Research Council of Canada	Partnership Grant	Large teams of postsecondary institutions and/or organizations of various types that work in formal collaboration.<br>Partner organizations can be Canadian or international institutions or organizations (public, private, not-for-	Applications must include:<br>• Relevant documentation to allow informed evaluation of the quality and level of commitment of the proposed formal partnerships.<br>• Evidence of formal partnership. Evidence can include, but is not limited to: Governance frameworks; Agreements (intellectual property, conflict resolution, etc.); Strategic plans; and Other relevant documentation.

		profit) of any type.	• A minimum of 35% in addition cash and/or in-kind contributions must be secured from partner organizations.
Société inclusive	Inclusive Society Partnership Research Program	Partnership between researcher and a non-profit organization that has a disability-related mission and a charitable number (e.g., a community-based organization).	Applications must include:<br>• Need of the partner or partners, background, and objectives.<br>• Roles and responsibilities of the partners.<br>• Methods of dissemination and appropriation of the project results by the researchers and by the partners.<br>• A letter from the principal partner explaining their need, the intended use of the results, and the number of people who will be affected by this research project. Partners also need to indicate their in-cash/in-kind contribution.
United States Department of Defense	Spinal Cord Injury Research Program: Clinical Trial Award	Collaborative research approaches are characterized by the equitable collaboration between community members and researchers. These collaborative relationships are often established through integrating community members into research teams as co-researchers, advisors, and/or consultants.	Research funded should be responsive to the needs of people with SCI, their families, and/or their care partners. Research teams are therefore required to establish and utilize effective and equitable collaborations and partnerships with community members to maximize the translational and impact potential of the proposed research.<br>Applications are expected to name at least two community partners (e.g., SCI Lived Experience Consultant, representative of community-based organization) who will provide advice and consultation throughout the planning and implementation of the research project.<br>Applications must include:<br>• Collaborative Research Statement<br>o Describe the collaborative research approach that will be used (e.g., Lived Experience Consultation, partnership with community-based organization, community advisory board, co-researcher model) including a justification for the approach as well as when the approach will be used within the research project.<br>o Indicate the input from the community partner that has or will be captured and how this input has and/or will be meaningfully integrated and incorporated into the needs assessment, planning, design, execution, analysis, and/or dissemination of the research.<br>o Detail the resource allocation and decision-making processes to be employed.<br>o Describe any training that will be provided to both scientific researchers and

	community members on collaborative research approaches, decision-making, and equitable participation.<br>○ Describe co-learning and capacity-building activities among all partners.<br>○ Outline the process measures to assess the effectiveness of the chosen collaborative research approach.<br>• A letter of community collaboration:<br>○ Provide a letter signed by each community partner (e.g., SCI Lived Experience Consultant; representative of community-based organization) confirming their role and commitment to participate on the research team.

Note: SCI – Spinal Cord Injury; IKT – Integrated Knowledge Translation.
