# Supplementary material for: Funding Programs Relevant to Spinal Cord Injury Research and Their Approaches to Research Partnerships: An Environmental Scan
Source: Int J Health Policy Manag. 2026 Apr 11;15:8813. doi: 10.34172/ijhpm.8813 (PMC13338737; doi:10.34172/ijhpm.8813)
Supplement: Supplementary file 5 — Program Information on Partnership Evaluation, Support, and Post-grant Accountability. [file ijhpm-15-8813-s005.pdf]

Article title: Funding Programs Relevant to Spinal Cord Injury Research and Their Approaches to Research Partnerships: An Environmental Scan

Journal name: International Journal of Health Policy and Management (IJHPM)

Authors' information: Zhiyang Shi[1], Alanna Shwed[2], Ian D. Graham[3], Gayle Scarrow[4], Peter Athanasopoulos[5], Vanessa K. Noonan[6], John Chernesky[6], Kathryn M. Sibley[7]*, SCI IKT Guiding Principles Partnership Panel#, Heather L. Gainforth[1]*

[1]Department of Kinesiology and Physical Education, McGill University, Montreal, QC, Canada.

[2]School of Health and Exercise Sciences, University of British Columbia Okanagan, Kelowna, BC, Canada.

[3]School of Epidemiology and Public Health, University of Ottawa, Ottawa, ON, Canada.

[4]Michael Smith Health Research BC, Vancouver, BC, Canada.

[5]Spinal Cord Injury Ontario, Toronto, ON, Canada.

[6]Praxis Spinal Cord Institute, Vancouver, BC, Canada.

[7]Department of Community Health Sciences, University of Manitoba, Winnipeg, MB, Canada.

#A full list of the investigators of the IKT Guiding Principles Partnership Panel is provided at the end of the article.

***Correspondence to:** Kathryn M. Sibley; Email: kathryn.sibley@umanitoba.ca & Heather L. Gainforth; heather.gainforth@ubc.ca

Citation: Shi Z, Shwed A, Graham ID, et al. Funding programs relevant to spinal cord injury research and their approaches to research partnerships: an environmental scan. Int J Health Policy Manag. 2026;15:8813. doi:10.34172/ijhpm.8813

Supplementary file 5. Program Information on Partnership Evaluation, Support, and Post-grant Accountability

Funding Agency	Funding Program	Evaluation on Partnership	Support for Partnership	Post-grant Evaluation
Canada Foundation for Innovation	College Fund	The institution has demonstrated its ability to build and maintain productive partnerships with an industry or community. The institution has identified partners for the planned applied research activities. The institution has a plan to stimulate new partnerships with the industry or community.	Not found.	Not found.
Canadian Institutes of Health Research	Strategy for Patient-Oriented Research (SPOR)	Extent to which patients, including PWLE [people with lived experience], and other knowledge users are meaningfully and equitably engaged throughout the project. Appropriateness of Patient Engagement Plan and the extent to which approaches align with the SPOR Patient Engagement Framework.<br><br>Extent of the role(s) and contribution(s) of all applicant partner(s) in advancing research objectives (if applicable), and consideration of SPOR-funded grantees and other Canadian Institutes of Health Research grantee collaborators.<br><br>Quality of proposed partnerships and anticipated sustainability of those partnerships.<br><br>Proactive and meaningful consideration of partnership risks, including the extent of real and/or perceived conflict of interest and appropriateness of its management and mitigation (if applicable).	Eligible expenses include:<br>• Compensation to patient partners (including PWLE).<br>• Support for research and collaborators outside of Canada as part of the funded trial.<br>• Expenditures that respect the culture and traditions of Indigenous peoples, where needed for the meaningful conduct of research.<br>• Release Time Allowance to facilitate the participation of knowledge user(s) in innovative Clinical Trial (iCT) projects.	Not found.
Craig H. Neilsen Foundation	SCI Research on the Translational Spectrum (SCIRTS) Senior Research	Not found.	Eligible expenses include:<br>• Accommodations for people with disabilities.	Require a final report [not specific to partnership].

	Grants			
Fonds de recherche du Québec	FRQ Public-Private Partnership Research Chairs program	Quality of the partnership:<br>• Integration, complementarity, and involvement of the expertise around the program.<br>• Dynamic relationship between the industry partners, researchers, students and postdoctoral fellows, and opportunities for skills enhancement related to partner needs.<br>• Exemplary nature of the proposed governance model.<br>• Level of engagement of the institution and industry partner(s).<br>Democratization of knowledge:<br>• Mechanisms put in place for dissemination, awareness and knowledge uptake among industrial partners, user communities, government members and the general public in an open science context.<br>Research benefits for innovation:<br>• Alignment of the program with community and partner needs.<br>• Anticipated knowledge and innovation benefits for the industry partner(s).	Not found	Partner contributions: An annual financial report of partner contributions must be submitted.<br>Partner assessment: After three years of funding, partners must submit a document indicating their assessment of the progress of the research activities.<br>The governance model proposed for the C3P should include follow-up committees with the industry partner(s), on which the FRQ will not sit.
Michael Smith Health Research BC	Convening & Collaborating Program (C2)	Team quality and engagement account for 50%:<br>• The roles of all team members are described, and the team has the expertise and experience to carry out the convening and collaborating activities.	Eligible expenses include:<br>• Partial or full salary support for research users’ participation as a team member or key meeting participant, including buy-out/release time from work.<br>• Cost of caregiving services (e.g., childcare)	Require a final report, which may include:<br>• A description of the involvement of trainees and research users with your team.

		• The convening and collaborating activities are collaboratively planned and implemented by the researcher and research user in a way that meaningfully engages the expertise of each individual.	to allow research users or key meeting participants to participate in virtual meetings.<br>• Costs for the purchase or maintenance of equipment required to bring people together for the purposes of collaborating, networking, and knowledge exchange (e.g., tablets, cell phones, etc.).<br>• Direct costs associated with bringing people together for the purposes of collaborating, networking, and knowledge exchange, including planning, co-ordination, translation, and/or outreach activities (e.g., teleconference call, internet conferencing, etc.).<br>• Travel to attend collaborative trips and similar meetings integral to the activity, program, or meeting(s) proposed in the C2 application.<br>All applicants will receive reviewers' comments on the applications.<br>An application example is provided on the website.	
Michael Smith Health Research BC	Reach Program	Same as C2.	Same as C2.	Same as C2.
Mitacs	Accelerate Program	Not found	Offers a one-hour webinar "Research Partnership 101s".<br>Local business development representative build connections between researchers and non-for-profit organizations and help review applications.	Require a final report [not specific to partnerships].
National Institute on Disability,	Spinal Cord Injury Model System Multi-	Input of individuals with disabilities and other key stakeholders is used to shape the	Not found.	Require an annual and a final report [not

Independent Living and Rehabilitation Research (U.S.)	Site Collaborative Research Project	proposed research activities.<br><br>The extent to which the applicant's proposed collaboration with one or more agencies, organizations, or institutions is likely to be effective in achieving the relevant proposed activities of the project.<br><br>The extent to which agencies, organizations, or institutions demonstrate a commitment to collaborate with the applicant.<br><br>The extent to which the applicant clearly documents its capacity to carry-out a multi-site research project, including demonstrated administrative capabilities, experience with managing and following multi-site research protocols, and ability to maintain and meet standards for quality and confidentiality of data gathered from multiple sites.		specific to partnership].
National Institutes of Health (U.S.)	NIH Research Project Grant	Not found.	Not found.	When multiple years are involved, recipients will be required to submit the Research Performance Progress Report annually and financial statements.<br><br>A final Research Performance Progress Report, invention statement, and the expenditure data portion of the Federal Financial Report are required for closeout of an award.<br><br>[none specific to partnership]
National Science Foundation (U.S.)	Partnerships for Innovation – Research Partnership (PFT-RP) track	The merits and appropriateness of the proposed partnership, and its role in catalyzing the technical, commercialization and educational objectives of the project.<br><br>The commitment of the proposed partners in reaching the stated goals of the proposal.	Not found.	During the course of the award, NSF may organize a PI grantee meeting, reverse site visits, or a convening between PFI teams, investors and industry representatives. Proposers should budget travel for the PI and one student or postdoctoral researcher to attend.<br><br>Based on project progress, grantees in the

		The commitment of the Industrial Partner to the commercialization of the technology beyond the term of the PFI award		PFI-RP track may be requested to present a project status update via a webinar format to the NSF program officer and other NSF staff between 12 and 18 months after the start of the award [none specific to partnership].<br><br>Require an annual and a final report [none specific to partnership].
Natural Sciences and Engineering Research Council of Canada	Accelerate Program	Not found	Offers a one-hour webinar “Research Partnership 101s”.<br><br>Local business development representative build connections between researchers and non-for-profit organizations and help review applications.	Require a final report [not specific to partnerships].
Office des personnes handicapées du Québec	Programme de subventions à l’expérimentation	Not found.	Not found.	The grant is paid as follows:<br><br>50% upon signing the agreement.<br><br>30% upon receipt of the progress report.<br><br>20% upon receipt of the final report and its summary.
PRAXIS Spinal Cord Institute	Consumer Program	Not found	Connect researchers who want to meaningfully and respectfully engage people with lived experience in their research as participants or partners in research.<br><br>Champion integrated Knowledge Translation (iKT) and apply the principles of iKT by involving knowledge users including people with lived experience in projects.<br><br>Leverage existing external capacity building opportunities (webinars, workshops, online platforms) to enhance knowledge and skills of researchers in meaningful engagement of	Not found

			people with lived experience.<br><br>Support opportunities to increase knowledge and awareness of evidence-informed SCI resources among people with lived experience to improve self-management and informed decision-making	
Rick Hansen Foundation	International Collaboration on Repair Discoveries (ICORD) Seed Grants	Not found.	Serve as a platform to connect researchers, research users, clinicians, and organizations from multiple disciplines.	Require an interim report and a final report [not specific to partnerships].
Social Sciences and Humanities Research Council of Canada	Partnership Grant	• Quality and genuineness of the formal partnership and associated management and governance arrangements and leadership, including involvement of partner organizations and others in the design and conduct of the research and/or related activities;<br>• Expertise of the team and appropriateness of partner organizations in relation to the proposed project;<br>• Quality of the equity, diversity and inclusion plan for promoting a diverse team, inclusive working environment and equitable opportunities within the partnership;<br>• Indications of other planned resources, including leveraging of cash and in-kind support from the host institution and/or from partner organizations; and<br>• Quality and appropriateness of the knowledge mobilization plans, including effective dissemination, exchange and engagement with stakeholders within and/or beyond the research community, where applicable.	The grants support both existing and new partnerships.<br><br>Applications successful in Stage 1 are awarded grants valued at up to $20,000. These funds help applicants prepare for the Stage 2 application.<br><br>A salary research allowance can be requested for not-for-profit organizations involved in the partnership with an applicant, co-director or co-applicant on the team to release them from duties to their organizations.	Grant holders will be expected to report on the use of grant funds, on funded activities undertaken during the grant period, and on outcomes [not specific to partnership].

		• Evidence of other knowledge mobilization activities (e.g., films, performances, commissioned reports, knowledge syntheses, experience in collaboration / other interactions with stakeholders, contributions to public debate and the media) and of impacts on professional practice, social services and policies, etc.;<br>• Past experience in formal partnerships.		
Société inclusive	Inclusive Society Partnership Research Program	The actual participation of the partners throughout the project:<br>• Methods of participation of the partners and involvement of the principal partner.<br>• Relevance of the project to the lead partner.<br>• Contribution in cash/in kind.<br>• Participation in knowledge transfer and dissemination of research results within the extended networks.<br>Anticipated impact in terms of improved social inclusion, knowledge transfer, and expected ownership by the partner(s):<br>• Potential impact on social inclusion (immediate or expected).<br>• Realistic dissemination plan with an impact on social inclusion.<br>• Appropriation of the planned knowledge by the partner and its network.<br>• Potential impact on future collaborations and grant applications and relevance of knowledge transfer strategies.	Participants who intend to submit a proposal can benefit from the help of an intersectional collaboration agent to connect with other researchers or partners.<br>Eligible expenses include:<br>• At least $10,000 in support of the partner(s) for their participation in the project and for the appropriation of the results.	Require regular follow-ups with an intersectional collaboration agent.<br>A final report [not specific to partnership].

United States Department of Defense	Spinal Cord Injury Research Program: Clinical Trial Award	Patient impact:<br>• How well the input of the community partner (e.g., SCI Lived Experience Consultant, representative of community-based organization) will be captured and to what extent this input will be meaningfully integrated and incorporated into the needs assessment, planning, design, execution, analysis, and/or dissemination of the research.<br>Personnel and Communication:<br>• To what degree the qualifications and background of the community partners (e.g., SCI Lived Experience Consultant, representative of community-based organization) are relevant to their roles within the team and to the proposed research project.<br>• Whether the levels of effort of the study team members are appropriate for successful conduct of the proposed trial.	Application guideline provides examples to explain the three types of partnership approaches:<br>• Lived Experience Consultation:<br>○ The research team includes at least one project advisor with lived SCI experience who will provide advice and consultation throughout the planning and implementation of the research project. Lived Experience Consultants may include individuals with SCI, their family members, or care partners.<br>• Partnership with a community-based organization:<br>○ The research team establishes partnerships with at least one community-based organization that provides advice and consultation throughout the planning and implementation of the research project. Community-based organizations may include advocacy groups, service providers, policy makers, or other formal organizational stakeholders.<br>• Community Advisory Board:<br>○ A community advisory board is composed of multiple community stakeholders and can take many forms, from a board of Lived Experience Consultants to a coalition of community-based organizations or any combination thereof. As with Lived Experience Consultants and organizational partners, the community advisory board provides advice and consultation throughout planning and	Require quarterly, and annual progress reports, as well as a final progress report [not specific to partnership].

implementation of the research project.

Note: SCI – Spinal Cord Injury.
